# Detecting asymptomatic recurrence after radical nephroureterectomy contributes to better prognosis in patients with upper urinary tract urothelial carcinoma

**DOI:** 10.18632/oncotarget.23982

**Published:** 2018-01-04

**Authors:** Hirotaka Horiguchi, Shingo Hatakeyama, Go Anan, Yuka Kubota, Hirotake Kodama, Masaki Momota, Koichi Kido, Hayato Yamamoto, Yuki Tobisawa, Tohru Yoneyama, Takahiro Yoneyama, Yasuhiro Hashimoto, Takuya Koie, Hiroyuki Ito, Kazuaki Yoshikawa, Toshiaki Kawaguchi, Makoto Sato, Chikara Ohyama

**Affiliations:** ^1^ Department of Urology, Hirosaki University Graduate School of Medicine, Hirosaki, Japan; ^2^ Department of Urology, Tohoku Medical and Pharmaceutical University, Sendai, Japan; ^3^ Department of Advanced Transplant and Regenerative Medicine, Hirosaki University Graduate School of Medicine, Hirosaki, Japan; ^4^ Department of Urology, Aomori Rosai Hospital, Hachinohe, Japan; ^5^ Department of Urology, Mutsu General Hospital, Mutsu, Japan; ^6^ Department of Urology, Aomori Prefectural Central Hospital, Aomori, Japan

**Keywords:** radical nephroureterectomy, asymptomatic, symptomatic, recurrence, upper tract urothelial carcinoma

## Abstract

**Background:**

The prognostic benefit of regular follow-up to detect asymptomatic recurrence after radical nephroureterectomy (RNU) remains unclear. We aimed to assess whether regular follow-up to detect asymptomatic recurrence after RNU improves patient survival.

**Materials and Methods:**

We retrospectively analysed 415 patients who underwent RNU for upper tract urothelial carcinoma at four hospitals between January 1995 and February 2017. All patients had regular follow-up examinations after RNU including urine cytology, blood biochemical tests, and computed tomography. We investigated the first site and date of tumor recurrence. Overall survivals of patients who developed recurrence, stratified by mode of recurrence (asymptomatic vs. symptomatic group), were estimated using the Kaplan–Meier method with the log–rank test. Cox proportional hazards regression analysis was performed using inverse probability of treatment weighting (IPTW) to evaluate the impact of the mode of recurrence on survival.

**Results:**

Of the 415 patients, 108 (26%) experienced disease recurrences after RNU. Of these, 62 (57%) were asymptomatic and 46 (43%) were symptomatic at the time of diagnosis. The most common recurrence site and symptom were lymph nodes and pain, respectively. Overall survival after RNU and time from recurrence to death in the asymptomatic group were significantly longer than that in the symptomatic group. Multivariate Cox regression analysis showed that symptomatic recurrence was an independent risk factor for overall survival after RNU and survival from recurrence to death.

**Conclusions:**

Routine oncological follow-up for detection of asymptomatic recurrence contributes to a better prognosis after RNU.

## INTRODUCTION

Upper tract urothelial carcinoma (UTUC) is a relatively rare disease, and prognosis of patients with advanced UTUC has not improved over the past two decades [1, 2]. Radical nephroureterectomy with bladder cuff excision (RNU) remains the standard treatment modality for UTUC patients, and regular oncological surveillance after RNU is necessary. The rationale for regular oncological surveillance is to detect tumor recurrence at an early stage so that it can be cured or at least treated with a better prognosis. The impact of regular surveillance on the long-term prognosis has been investigated in the context of several malignancies. However, most studies found no survival benefit accruing from regular follow-up of patients with colorectal [3, 4], breast [5], endometrial [6], and lung cancers [7]. Similarly, the impact of regular surveillance on prognosis continues to be debated in urinary bladder cancer. Till date, only five studies have investigated the benefit of routine oncological follow-up; their conclusions suggest the benefit of routine oncological follow-up [8–12]. However, due to the lack of robust evidences, it remains controversial. In UTUC, there is no evidence to prove if routine oncological follow-up after RNU to detect asymptomatic recurrence improves patient survival. Because a prospective randomized study to clarify this issue is not feasible, use of statistical methods, to eliminate the confounding influences introduced by nonrandom treatment assignment in retrospective studies, is a key imperative. In this study, we compared the impact of mode of diagnosis (asymptomatic vs. symptomatic) of recurrence on patient survival after RNU and survival after recurrence, using an inverse probability of treatment weighting (IPTW) strategy.

## RESULTS

### Baseline characteristics

**Figure 1:**
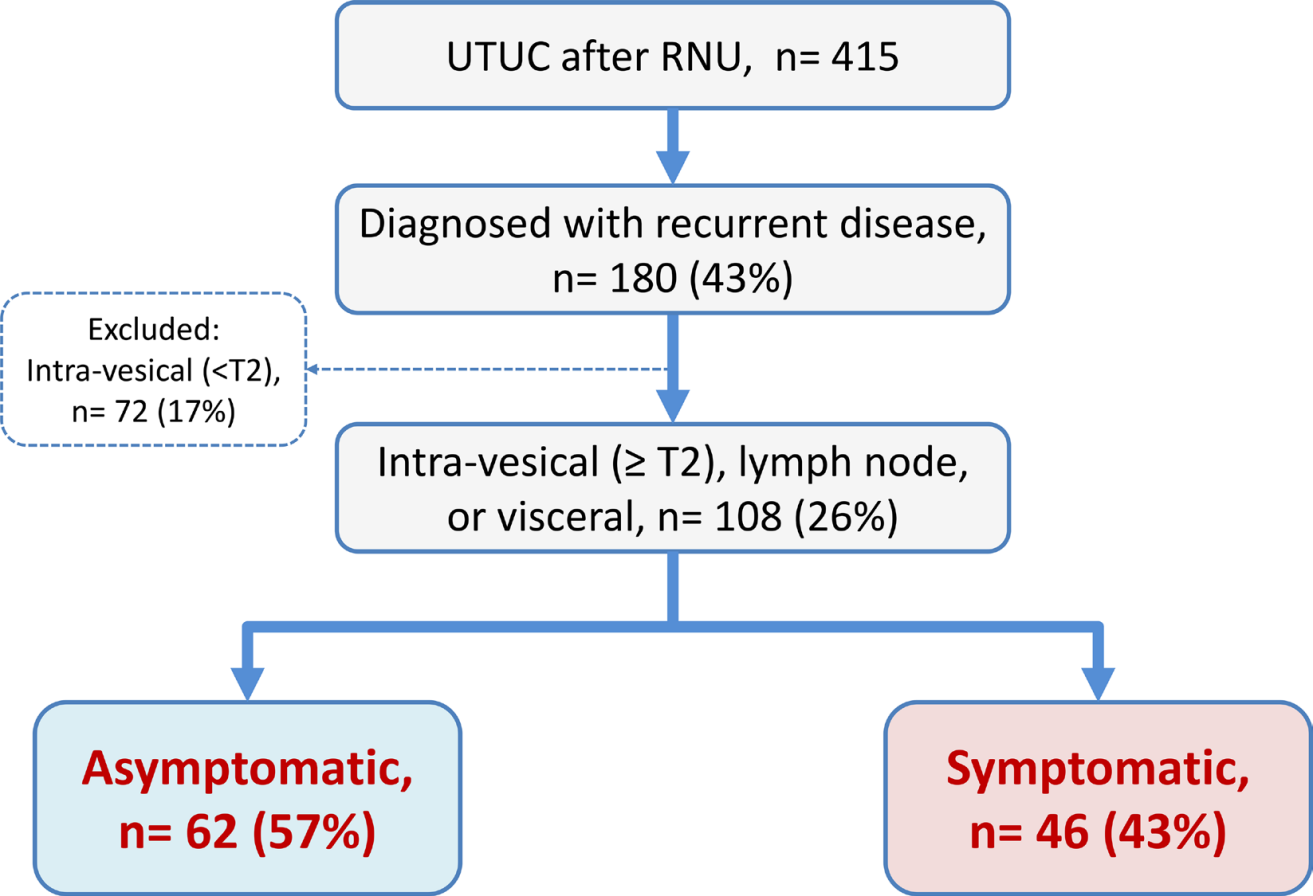
Patient selection Of 108 patients with disease recurrence, 62 (57%) were detected during regular follow-up examinations in an asymptomatic state, whereas in 46 (43%) patients, recurrence was detected by symptom-driven examinations.

Of the 415 patients, 180 (43%) developed disease recurrence over a median follow-up duration of 42 months. Of these, 72 patients (17%) who had intra-vesical (< T2) recurrence alone were excluded from the present study. Out of 108 patients, 62 (57%) were asymptomatic at the time of recurrence detection during the regular follow-up examination (asymptomatic group), whereas in 46 (43%) patients, recurrence was detected by symptom-driven investigations (symptomatic group) (Figure 1). Clinicopathological characteristics of patients disaggregated by study group are presented in Table 1. Except for neoadjuvant chemotherapy, surgical margin, and chemotherapy recieved after recurrence, no significant differences were observed with respect to patient background or tumor-related variables between the groups. The frequency of chemotherapy after recurrence was significantly different between in the asymptomatic (77%) and symptomatic groups (57%) (*P* = 0.024). In addition, the median number of chemotherapy courses after recurrence were significantly different between in the asymptomatic (3 ± 4) and symptomatic groups (2 ± 3) (*P* = 0.040) (Table 1).

**Table 1:** Background of patients

	All	Asymptomatic	Symptomatic	*P* value
*n*	415	62	46	
Age (years)	70 ± 8.9	71 ± 9.2	71 ± 9.8	*0.935*
Gender (Male), *n =*	287 (69%)	41 (66%)	29 (63%)	*0.806*
ECOG-PS > 1, *n =*	10 (2.4%)	2 (3.2%)	3 (6.3%)	*0.649*
Hypertension (HTN), *n =*	181 (44%)	28 (45%)	16 (35%)	*0.242*
Diabetes mellitus (DM), *n =*	69 (17%)	9 (15%)	4 (10%)	*0.327*
Cardiovascular disease (CVD), *n =*	76 (18%)	5 (8.1%)	10 (22%)	*0.052*
Smoking, n=	193 (47%)	27 (44%)	18 (39%)	*0.537*
eGFR before surgery (ml/min/1.73 m^2^)	58 ± 19	51 ± 13	48 ± 12	*0.640*
Neoadjuvant chemotherapy (NAC), *n =*	100 (24%)	10 (16%)	16 (35%)	*0.039*
TNM classification, *n* =				
cT3 or 4	222 (54%)	45 (73%)	36 (78%)	*0.654*
cN+	34 (8.2%)	5 (8.1%)	2 (4.3%)	*0.432*
Original tumor sites, *n =*				
Renal pelvis	165	18	9	
Ureter	220	39	31	*0.607*
Multiple	30	6	5	
Laparoscopic surgery, *n =*	71 (17%)	6 (9.7%)	2 (4.3%)	*0.462*
Postoperative complications, *n =*				
G3 or higher	14 (3.4%)	1 (1.6%)	4 (8.7%)	*0.161*
Pathological outcomes, *n =*				
pT3 or 4	179 (43%)	46 (26%)	32 (30%)	*0.666*
pN+	30 ‘7.2%)	9 (15%)	8 (17%)	*0.727*
High grade	179 (43%)	62 (100%)	46 (100%)	*1.000*
Surgical margin (SM) positive	14 (3.4%)	3 (4.8%)	9 (20%)	*0.027*
Lymphovascular invasion (LVI)	125 (30%)	33 (53%)	32 (70%)	*0.086*
Chemotherapy received after recurrence, *n* =				
Underwent	74 (13%)	48 (77%)	26 (57%)	*0.024*
Number of courses	2 ± 3	3 ± 4	2 ± 3	*0.040*
Deceased, *n =*	102 (25%)	41 (66%)	37 (80%)	*0.101*
Median follow-up (Months)	42	31	21	*0.057*

ECOG PS: Eastern Cooperative Oncology Group Performance Status.

### Characteristics of recurrence

**Figure 2:**
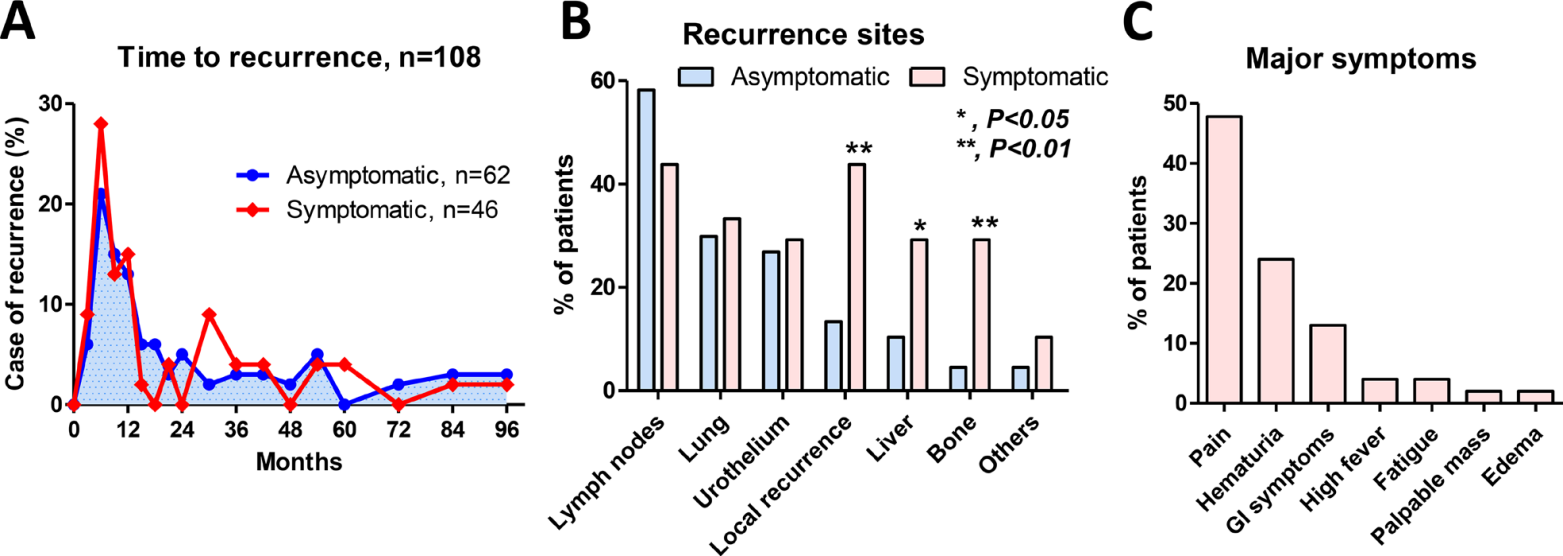
Characteristics of recurrence Overall, 80 patients (74%) experienced tumor recurrence within 24 months. Time course of recurrence within 24 months was not significantly different between the asymptomatic (47/62, 76%) and symptomatic (33/46, 70%) groups (*P* = 0.633) (**A**). The local recurrence, liver, and bone were significantly more frequent sites of recurrence in the symptomatic group (**B**). The most common symptom reported among the 46 patients who experienced symptomatic recurrence was pain (*n* = 22, 49%) followed by hematuria (*n* = 11, 23%) (**C**).

Of the 108 patients who developed tumor recurrence, 80 (74%) experienced tumor relapse within 24 months. Time course of recurrence within 24 months was not significantly different between the asymptomatic (47/62, 76%) and symptomatic (33/46, 70%) groups (*P* = 0.633; Figure 2A). Lymph nodes were the most common site of recurrence in both groups. Local recurrence, liver, and bone were significantly more frequent sites of recurrence in the symptomatic group (Figure 2B). The most common symptom reported in the symptomatic group was pain (*n* = 22, 49%) followed by hematuria (*n* = 11, 23%) (Figure 2C).

### Oncological outcomes

**Figure 3:**
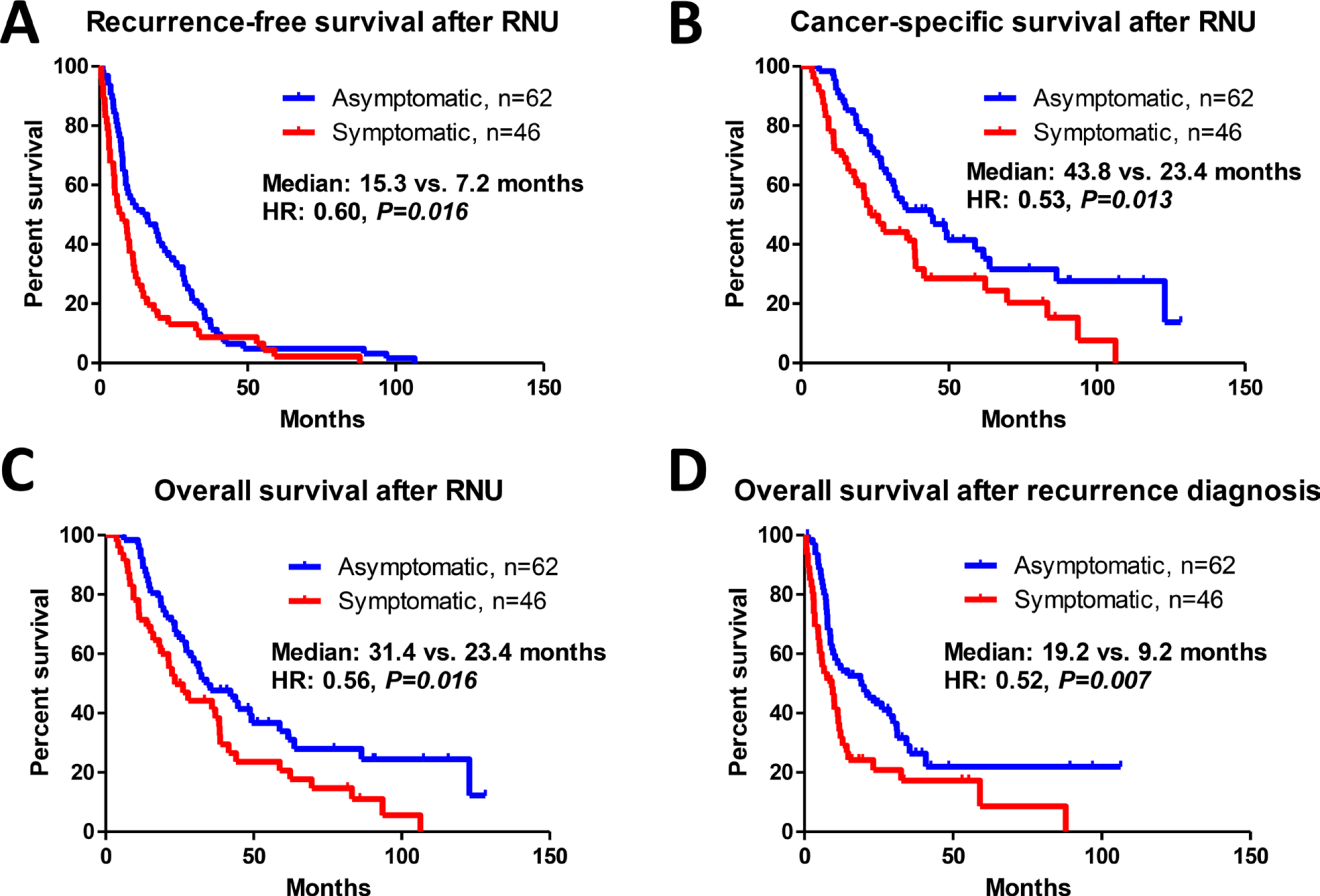
Prognostic assessment of patients with asymptomatic and symptomatic recurrence Recurrence-free survival (**A**), cancer-specific survival (**B**), and overall survival (**C**) after radical nephroureterectomy (RNU) were significantly longer in the asymptomatic group than that in the symptomatic group. Similarly, overall survival after recurrence in the asymptomatic group was significantly longer than that in the symptomatic group (**D**).

**Figure 4:**
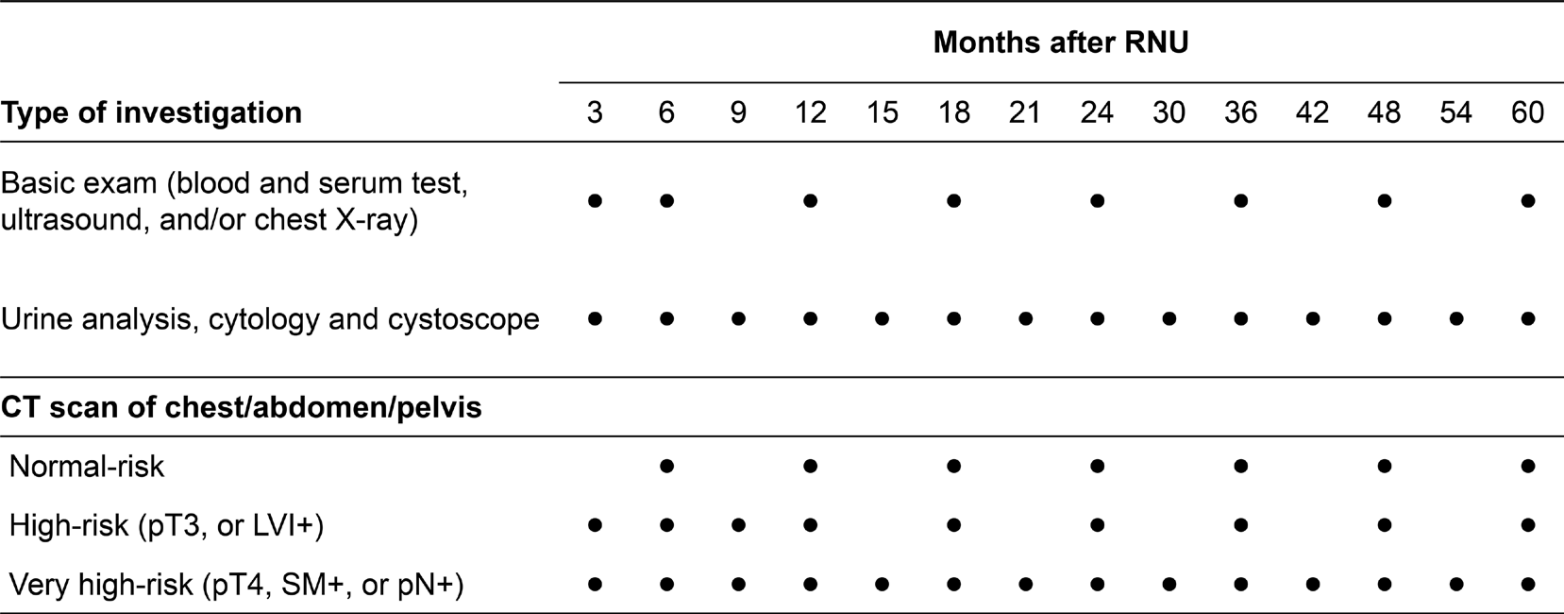
Standard protocol for surveillance after radical nephroureterectomy (RNU) Based on the pathological outcomes after radical nephroureterectomy, patients were divided into three groups (normal-risk, high-risk, and very high-risk) for risk stratification. Further investigations such as bone scans were ordered when clinically indicated. High-risk: pT3, positive lymphovascular invasion (LVI+); very high-risk: pT4, positive surgical margin (SM+), or pathological lymph node involvement (pN+). ●: scheduled examination.

**Figure 5:**
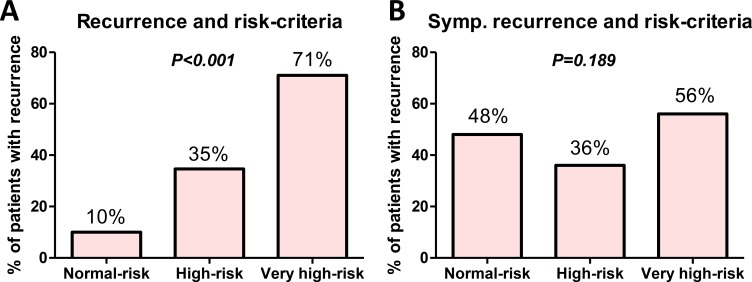
Relationship between mode of recurrence and risk-criteria The impact of risk stratification on mode of recurrence was investigated. The number of patients with the normal-, high-, and very high-risk were 202 (49%), 178 (43%), and 35 (8.4%), respectively. The number of patients with disease recurrences in the normal-, high-, and very high-risk were 21 (10%), 62 (35%), and 25 (71%), respectively (*P* < 0.001) (**A**). The number of patients with symptomatic recurrences in the normal-, high-, and very high-risk were 10 (48%), 22 (36%), and 14 (56%), respectively (*P* = 0.189) (**B**).

Among the 108 patients who developed recurrence, the number of deaths due to cancer and deaths from any cause were 70 and 78, respectively. The number of deaths due to cancer in the asymptomatic and symptomatic groups were 36 (58%) and 34 (76%), respectively. The number of deaths from any cause in the asymptomatic and symptomatic groups were 41 (66%) and 37 (80%), respectively. Recurrence-free survival (*P* = 0.016; Figure 3A), cancer-specific survival (*P* = 0.013; Figure 3B), and overall survival (*P* = 0.016; Figure 3C) after RNU were significantly longer in patients in the asymptomatic group than those in the symptomatic group. In addition, overall survival after recurrence in the asymptomatic group was significantly longer than that in the symptomatic group (*P* = 0.007; Figure 3D). Our surveillance protocol was shown in the Figure 4. Based on this, we investigated the impact of risk stratification on mode of recurrence. The number of patients with the normal-, high-, and very high-risk were 202 (49%), 178 (43%), and 35 (8.4%), respectively. The number of patients with disease recurrences in the normal-, high-, and very high-risk were 21 (10%), 62 (35%), and 25 (71%), respectively (*P* < 0.001; Figure 5A). The number of patients with symptomatic recurrences in the normal-, high-, and very high-risk were 10 (48%), 22 (36%), and 14 (56%), respectively (*P* = 0.189; Figure 5B). Although the frequencies of disease recurrence were significantly different, there were no difference in the number of symptomatic recurrence among the risk criteria.

### Uni- and multivariate Cox proportional hazards regression analyses

Univariate Cox regression analyses showed that symptomatic recurrence was a significant risk factor for post-RNU (*P* = 0.017) and post-recurrence overall survival (*P* = 0.008). On multivariate Cox regression analysis using IPTW, symptomatic recurrence was found to be an independent risk factor for post-RNU (*P* = 0.040) and post-recurrence overall survival (*P* = 0.009) (Table 2). The crude models of multivariate Cox regression analyses were shown in the Table 3.

**Table 2:** Uni- and multivariate Cox regression analyses for prognosis

Radical nephroureterectomy to death		Risk factor	*P* value	HR	95% CI
Univariate	Mode of recurrence	Symptomatic	*0.017*	1.73	1.10–2.71
Model 1 (Age, sex, NAC adjusted)		Symptomatic	*0.019*	1.76	1.10–2.84
Model 2 (Model 1 + pT, pN, LVI, and SM adjusted)		Symptomatic	*0.048*	1.70	1.01–2.88
IPTW^*^ model		Symptomatic	*0.040*	1.75	1.03–2.99
**Recurrence to death**		**Risk factor**	***P* value**	**HR**	**95% CI**
Univariate	Mode of recurrence	Symptomatic	*0.008*	1.83	1.17–2.87
Model 1 (Age, sex, NAC adjusted)		Symptomatic	*0.002*	2.14	1.32–3.46
Model 2 (Model 1 + pT, pN, LVI, and SM adjusted)		Symptomatic	*0.002*	2.26	1.34–3.83
IPTW^*^ model		Symptomatic	*0.009*	2.08	1.20–3.58

^*^, including age, sex, ECOG PS, smoking, cardiovascular disease, diabetes mellitus, eGFR, tumor location, neoadjuvant chemotherapy (NAC), postoperative complication (G3 or higher), pT, pN, lymphovascular invasion (LVI), surgical margin (SM), chemotherapy after recurrence. IPTW: inverse probability of treatment weighting.

**Table 3:** Crude model of multivariate Cox regression analyses for prognosis

Radical nephroureterectomy to death	Factor	*P* value	HR	95% CI
Age	Continuous	*0.950*	0.98	0.60–1.61
Gender	Male	*0.976*	1.00	0.97–1.03
Preoperative eGFR	Continuous	*0.649*	1.01	0.98–1.03
Neoadjuvant chemotherapy (NAC)	Underwent	*0.357*	1.38	0.70–2.71
pT	3 or 4	*0.047*	1.88	1.01–3.49
pN	Positive	*0.723*	1.12	0.60–2.08
LVI	Positive	*0.119*	1.59	0.89–2.84
Mode of recurrence	Symptomatic	*0.041*	1.69	1.02–2.80
**Recurrence to death**	**Factor**	***P* value**	**HR**	**95% CI**
Age	Continuous	*0.711*	0.91	0.57–1.47
Gender	Male	*0.751*	1.00	0.97–1.02
Preoperative eGFR	Continuous	*0.496*	0.99	0.97–1.02
Neoadjuvant chemotherapy (NAC)	Underwent	*0.355*	0.73	0.37–1.43
pT	3 or 4	*0.051*	1.83	1.00–3.35
pN	Positive	*0.760*	1.10	0.59–2.06
LVI	Positive	*0.961*	1.01	0.57–1.80
Mode of recurrence	Symptomatic	*0.003*	2.21	1.32–3.70

## DISCUSSION

In the present study population, 43% of patients presented with symptoms at recurrence. We found that patients with symptomatic recurrence had a significantly worse prognosis than those with asymptomatic recurrence. Patients with symptomatic recurrence more frequently experienced local recurrence, liver, and bone metastases. These findings are consistent with those of our previous study on symptomatic recurrence after radical cystectomy [12]. Recurrence-free survival, cancer-specific survival after RNU, and overall survival after recurrence were significantly longer in patients in the asymptomatic group than those in the symptomatic group. In addition, multivariate analyses using IPTW revealed that the presence of symptoms at recurrence was a significant predictor of poor overall survival after RNU (HR: 1.75) and after recurrence diagnosis (HR: 2.08). However, it was difficult to eliminate the possibility of the presence of symptoms that depend on a late diagnosis (lead-time bias). In addition, obvious tumor progression that is missed during routine follow-up suggests the existence of biological heterogeneity between rapid- and slow-growing tumors that could not have been detected by a conventional biomarker and/or pathological examination. We found clear differences with respect to site of recurrence between patients with and without symptomatic recurrence. Symptomatic recurrences were more frequent in the local recurrence, liver, and/or bone. For a better understanding of tumor biology, not only the mode of diagnosis (asymptomatic vs. symptomatic) of recurrence but also biomarkers that predict the malignant potential are essential. The development of a biology-based classification is a key imperative, which can potentially improve the clinical management of UTUC.

The rationale for follow-up examinations is to detect tumor recurrence at a stage when these are amenable to cure or can at least be treated with a better prognosis. However, whether routine oncological follow-up to detect asymptomatic recurrence improves patient survival is subject to debate in the context of several malignancies [3–10]. Although clinical benefit of oncological follow-up after RNU is yet to be demonstrated, the benefits of routine surveillance have been reported in the context of bladder cancer. In a series of 1,270 patients who underwent radical cystectomy, Volkmer *et al.* found no difference in overall survival between patients with asymptomatic and symptomatic tumor recurrence [8]. Conversely, Giannarini *et al.* noted that of 479 patients who underwent radical cystectomy with orthotopic ileal neobladder reconstruction, those diagnosed with asymptomatic recurrence during routine follow-up had significantly improved cancer-specific and overall survival compared with patients diagnosed after symptomatic relapse [9]. Boorjian *et al.* reported prognostic benefits accruing from detection of asymptomatic recurrence in a series of 1,599 patients who underwent radical cystectomy [10]. Similar findings were reported more recently by Alimi *et al.* in a series of 331 patients who underwent radical cystectomy and also observed by us in our recent study of 581 patients who underwent radical cystectomy [11]. However, the results are far from conclusive, and it is difficult to draw definitive conclusions owing to lack of prospective studies and poor comparability of data from different countries and from different medical insurance systems.

Yafi *et al.* proposed a stage-based protocol for the surveillance of patients after radical cystectomy [13]. Their extensive examination of recurrence patterns in a large multi-institutional project emphasizes the need for earlier strict surveillance in patients with high-risk diseases. Optimal schedule and risk stratification are necessary to clarify the efficacy of routine oncological surveillance and to limit over-investigation of patients with urothelial carcinoma. We recently reported risk stratification-based surveillance protocol after radical cystectomy that improves cost effectiveness [14, 15]. In addition, our recent study suggested preoperative impaired renal function had significant impact of poor prognosis in the patients with UTUC [16]. Therefore, utility of regular follow-up examinations after surgery is next question need to address. Our next study will address the risk-based optimal surveillance protocol after RNU.

The present study had several limitations. First, it was a retrospective study with a small number of patients who developed recurrence. We could not control all variables including selection bias, the influence of lead-time bias, and other unmeasurable confounding factors. In addition, there were strong historical biases for the indication of NAC in the present study because we started NAC for UTUC after 2010. Although our previous study suggested clinical benefit of NAC for UTUC [17], no prospective study is available to reveal the benefit of NAC on mode of recurrences. Therefore, accumulation of evidence from well-planned studies is essential. Second, our results cannot be applied to other nations because the entire Japanese population is covered by universal health insurance (maximum copayment from 10% to 30%). Despite these limitations, this is the first study to investigate the clinical benefit of routine oncological follow-up after RNU. Our results support the idea that an effective follow-up protocol following curative surgery should detect recurrence in the early stages of the disease and that detection with asymptomatic recurrence should secure sufficient time to implement a multimodal therapy after relapse.

In conclusion, routine oncological follow-up for the detection of asymptomatic recurrence may potentially improve the prognosis of patients after RNU. Further investigation with a well-designed study is necessary to assess the survival benefit of a surveillance protocol for patients with UTUC.

## MATERIALS AND METHODS

### Ethics statement

This retrospective study was performed in accordance with the ethical standards of the Declaration of Helsinki and approved by the Ethics Committee at the Hirosaki University School of Medicine (authorization numbers: 2015–258 and 2016–225). The study participants provided their verbal informed consent, which was recorded in their medical charts. Pursuant to the provisions of the ethics committee and the ethical guidelines in Japan, written consent was not required for public disclosure of study-related information, such as that from the existing documentation, in the case of a retrospective and/or observational study.

### Patient selection

Between January 1995 and February 2017, 415 adult patients underwent RNU at Hirosaki University Hospital, Aomori Rosai Hospital, Mutsu General Hospital, and Aomori Prefectural Central Hospital. We stratified the patients into two groups based on mode of diagnosis of recurrence between the patients with asymptomatic recurrence (asymptomatic group) or with symptomatic recurrence (symptomatic group).

### Evaluation of variables

The analyzed variables were age, sex, Eastern Cooperative Oncology Group Performance Status (ECOG PS), history of cardiovascular disease (CVD), hypertension (HTN), diabetes mellitus (DM), smoking, clinical and pathological stage, postoperative complications, renal function, tumor sites, NAC, and mode of diagnosis of recurrence (asymptomatic vs. symptomatic). Postoperative complications within 30 days were evaluated based on the Clavien–Dindo classification [18]. Renal function was evaluated using the estimated glomerular filtration rate (eGFR). The following equation was used to estimate eGFR for Japanese patients; it is a modification of the abbreviated Modification of Diet in Renal Disease Study formula: eGFR mL/min/1.73 m^2^ = 194 × sCr^−1.094^ × age^−0.287^ (× 0.739, if female) [19]. Tumor stage and grade were assigned according to the 2009 TNM classification of the Union of International Cancer Control [20].

### Neoadjuvant chemotherapy (NAC)

Since September 2006, we have treated selected patients with two to four courses of NAC in advanced (cT3-4 and/or cN+) cases [17]. NAC is a platinum-based combination regimen comprising of either gemcitabine plus cisplatin (GCis), gemcitabine plus carboplatin (GCarbo), or methotrexate, vinblastine, adriamycin, and cisplatin (MVAC). Regimens were selected based on guidelines for the proper use of cisplatin [21] and on the patient’s overall status.

### Surgical procedure

Open or laparoscopic nephroureterectomy, which includes the removal of kidney, ureter, and ipsilateral bladder cuff excision, was performed [22]. The distal ureter was managed by the extravesical approach. A non-template-based regional lymph node dissection was performed. Postoperative complications were reviewed using the Clavien–Dindo classification.

### Follow-up protocol for surveillance

Oncological follow-up after RNU was performed according to the European Association of Urology guidelines [1] and the Japanese guidelines for UTUC [23] and bladder cancer [24]. Our follow-up protocol consisted of complete blood counts, serum chemistry screenings, ultrasound imaging of abdomen, computed tomography (CT), and chest radiography every 3–6 months for at least five years. Based on the pathological outcomes after RNU, patients were divided into three groups: very high-risk (pT4, surgical margin+ (SM+), or pathological lymph node involvement: pN+); high-risk (pT3 or positive lymphovascular invasion: LVI); and normal-risk. Our standard protocol for very high-risk patients recommends CT follow-up every three months for the first two years after surgery, semiannually for the next three years, and annually thereafter in patients without any evidence of disease recurrence. The CT protocol for high-risk patients recommends follow-up every three months for the first year, every six months for next year and annually thereafter in patients who have no evidence of disease recurrence. The CT protocol for normal-risk patients recommends follow-up every six months for the first two years and annually thereafter in patients who have no evidence of disease recurrence (Figure 4). Additional examinations such as a bone scan or brain imaging were performed when clinically indicated. A cystoscope protocol recommends cystoscope, urine cytology and urine analysis every three months for the first two years after surgery, semiannually for the next three years, and annually thereafter in patients who have no evidence of disease recurrence regardless of risk classification.

Disease recurrence was classified based on the involved site such as lymph nodes, visceral organs, local recurrence in the pelvis, bone, urothelium, or brain. Non-invasive, superficial urothelial recurrences were excluded from the present study. Lymph node recurrence included metastasis to local recurrence, para-aortic, thoracic, mediastinal, and paratracheal lymph nodes. Visceral organ recurrence included metastasis to the liver, lungs, adrenal glands, and other intra-abdominal organs. The first recurrence after RNU was recorded. We also analyzed whether the first tumor recurrence was detected solely based on imaging findings during the scheduled follow-up investigations in asymptomatic patients or whether the recurrence was diagnosed by symptom-driven investigations. The vital status was determined from death certificates or from physician’s correspondence.

### Adjuvant and salvage therapy

Adjuvant chemotherapy and/or radiotherapy were not routinely administered. Salvage therapy was indicated only when a visible tumor was identified. Systemic chemotherapy after recurrence consisted of a platinum-based combination regimen, including GCis, GCarbo, GCD (gemcitabine, carboplatin and docetaxel), DIN (docetaxel, ifosfamide, and nedaplatin), or MVAC. Regimens were selected based on residual renal function and the patient’s overall status.

### Statistical analysis

Statistical analyses were performed using SPSS version 24.0 (SPSS Inc., Chicago, IL, USA), GraphPad Prism 5.03 (GraphPad Software, San Diego, CA, USA), and R 3.3.2 (The R Foundation for Statistical Computing, Vienna, Austria). Categorical variables were compared using Fisher’s exact test or Chi-squared test. Between-group differences with respect to normally distributed variables were assessed using Student’s *t*-test; those with respect to non-normally distributed variables were assessed using Mann–Whitney *U* test. All tests were two-sided and a *P* value < 0.05 was considered statistically significant. Overall survival in the asymptomatic and symptomatic groups were calculated as time from RNU to date of death, and time from first recurrence to death from any cause, respectively, using Kaplan-Meier method. Between-group difference in overall survival was assessed with log rank test. Cox proportional hazards regression models were used to evaluate the impact of the mode of diagnosis of recurrence on survival, and hazard ratios (HRs) with 95% confidence intervals were calculated. Due to the limited sample size, we performed a multivariate Cox proportional hazards regression analysis using IPTW, which reweighs both the affected and unaffected groups to generate a propensity score-matched population [25], to evaluate the impact of symptom recurrence on survival (overall survival since the RNU and since the recurrence diagnosis). Variables included in the IPTW analysis were age, sex, ECOG PS, smoking, CVD, DM, eGFR, tumor location, NAC, postoperative complication grade, pT, pN, LVI, surgical margin, and chemotherapy received after recurrence.

## Abbreviations

RNU: radical nephroureterectomy
UTUC: upper tract urothelial carcinoma
IPTW: inverse probability of treatment weighting
ECOG PS: Eastern Cooperative Oncology Group Performance Status
CVD: cardiovascular disease
HTN: hypertension
DM: diabetes mellitus
eGFR: estimated glomerular filtration rate
NAC: neoadjuvant chemotherapy
GCis: gemcitabine plus cisplatin
GCarbo: gemcitabine plus carboplatin
MVAC: methotrexate, vinblastine, adriamycin, and cisplatin
LVI: lymphovascular invasion
SM: surgical margin
HR: hazard ratio
